# Effects of *Scleroderma sichuanensis* Xiao (Hymenoptera: Bethylidae) venom and parasitism on nutritional content regulation in host *Tenebrio molitor* L. (Coleoptera: Tenebrionidae)

**DOI:** 10.1186/s40064-016-2732-1

**Published:** 2016-07-08

**Authors:** Zhi-hang Zhuo, Wei Yang, Dan-ping Xu, Chun-ping Yang, Hua Yang

**Affiliations:** Provincial Key Laboratory of Forest Protection, College of Forestry, Sichuan Agricultural University, Wen’jiang City, 611130 Sichuan Province China; College of Food Science, Sichuan Agricultural University, Ya’an City, 625014 Sichuan Province China

**Keywords:** *Scleroderma sichuanensis* Xiao, *Tenebrio molitor* L. pupa, Venom, Parasitized, Nutritional content regulation

## Abstract

To explore the mechanisms by which the wasp *Scleroderma sichuanensis* Xiao regulates the physiology and biochemistry of its host, effects of *S. sichuanensis* venom and parasitism on host the *Tenebrio molitor* L. pupae were examined. Significant differences in nutritional content were noted between parasitized and non-parasitized pupae and between venom- and phosphate buffered saline-injected pupae. When pupae were injected with venom, the fat body could not be disintegrated into granules; however, when pupae were parasitized, fat-body disintegration occurred. Electrophoresis showed no differences in hemolymph protein content between parasitized pupae and those injected with venom, indicating that the wasp did not have narrow-spectrum peptides. These findings confirmed that *S. sichuanensis* was a typical idiobiont ectoparasitoid wasp, and that nutrient regulation was similar between idiobiont and koinobiont wasps. The strong similarities between the two treatments suggest that venom injection is a major factor responsible for changes in host nutrient content. The wasp fed mainly on reducing sugars, free amino acids, and fat-body tissues; larval fat bodies were derived from hemolymph and from host tissue. Our findings suggest that lipid catabolism might be accelerated, and that lipid biosynthesis might be inhibited, when host pupae are parasitized or injected with venom. In addition to venom, physiological and biochemical changes that occur during the parasitic process might be caused by venom, ovarian proteins, saliva, or secretions.

## Background

Parasitic wasp venom has many effects on hosts, including inhibition of the host immune and reproductive systems, regulation of growth and development, endocrine regulation and targeted gene expression (Nakamatsu et al. 2002; Nakamatsu and Tanaka 2003, 2004a, b, c; Zhang et al. 2004; Suzuki and Tanaka 2007; Kwon and Kim 2008; Nguyen et al. 2008; Martinson et al. 2014). For example, when *Pieris rapae* was parasitized by *Cotesia rubecula*, its hemolymph phenol oxidase (tyrosinase) activity was significantly reduced (Asgari et al. 2003). Cai et al. (2004) showed that venom of *Pteromalus puparum* could significantly inhibit the extension and encapsulation functions of hemocytes in *P. rapae* pupae, and could lead to hemocyte death. Webb and Luckhart (1994) suggested that venom and ovarian proteins of *Campoletis sonorensis* could inhibit the host’s early response by destroying the skeleton of host plasmatocytes and granulocytes. Moreover, the venom of *Nasonia vitripennis* accelerated host fat synthesis (Rivers and Denlinger 1995). For most parasitoids that lack polydnavirus (PDV) or teratocytes, venom is the most critical factor in regulating host physiology (Askew and Shaw 1986; Doury et al. 1997; Parkinson and Weaver 1999; Richards and Parkinson 2000; Dani et al. 2003; Cai et al. 2004; Li et al. 2006).

*Scleroderma sichuanensis* (Hymenoptera: Bethylidae) is a wasp that parasitizes many borer pests, such as *Callidium villosulum* Fairmaire, *Batocera horsfieldi* Hope, and *Monochamus alternatus* Hope, and that can cause permanent host paralysis (Zhou et al. 1997). *S. sichuanensis* also can regulate host nutritional content for its own benefit. For example, storage carbohydrates (glycogen and trehalose) and protein contents were decreased, free amino acids and reducing sugar contents were increased, and hydrolytic enzyme activity was significantly increased in hosts parasitized by *S. sichuanensis* (Tan and Zhou 2003; Tan et al. 2003). Furthermore, although female *S. sichuanensis* have a simple reproductive system and no distinct calyx, the volume of their poison sac is proportionally high compared with other organs; it indicates that venom plays an important role in the relationship between *S. sichuanensis* wasps and hosts (Jiang and Zhou 2006). Studies of the regulatory relationship between *S. sichuanensis* and its hosts have mostly focused on changes in the wasp, especially on fecundity, learning behavior and parasitization capacity; there are fewer reports about changes in host nutrition (Yang et al. 2005, 2007; Tan et al. 2003). Here, we examined host nutrient indicators (carbohydrates, lipids, and proteins) to assess the physiological mechanism by which *S. sichuanensis* regulates the nutritional content of its host, *T. molitor*.

## Materials and methods

### Materials

Adult female *S. sichuanensis* were reared on *T. molitor* pupa (0.1–0.15 g), which were reared on wheat bran and pollution-free vegetables, at the Provincial Key Laboratory of Forest Protection (College of Forestry, Sichuan Agricultural University, Sichuan Province, China). Wasps that had emerged for 6 days and completed mating were used in the experiments.

### Materials pre-treatment

Before the experiment, mated female wasps and host pupae (1:1 ratio) were placed in finger-shaped glass tubes (6 cm × 1 cm). This procedure was designed to ensure that the wasps were familiar with and efficient at injecting and paralysing hosts. If hosts could be paralysed within 3 h, we considered experienced to be successful. This familiarization helped to maintain equal-instars pupa in the treatment and control groups. Experienced wasps and pupae (1:1 ratio) were placed in finger-shaped glass tubes. The tube orifices were sealed by cotton wool, and the tubes were kept under natural light. When wasps successfully paralysed the host for 24 h, we defined it as 1 day.

### Preparation of venom and calculation of protein content

Venom sacs were carefully removed from the abdomens of female *S. sichuanensis* under a dissecting microscope and placed in 20 μL Dulbecco’s phosphate buffered saline (PBS: 0.80 % NaCl, 0.01 mol L^−1^ phosphate, pH 7.0–7.2) in the cap of an 0.5-mL centrifuge tube. The sac was then torn open to release the venom into the solution, and the empty sac was removed. After replacing the cap on the centrifuge tube, the tube was briefly centrifuged at 10,000×*g* and 4 °C (10 s), and the venom was collected and placed on ice. Venom from up to 20 wasps was collected in this way and pooled in 1 tube. The venom was then passed through a pipette tip to ensure thorough mixing, and centrifuged for 30 s (10,000×*g*, 4 °C) to pelletize; the supernatant was collected and stored at −20 °C. A standard curve was prepared using bovine serum albumin, and venom protein (0–100 μg) was quantified spectrophotometrically according to Bradford (1976). The calculation of protein content test was repeated five times by using 5 samples.

### Natural injection and venom injection

#### Parasitism (natural injection)

Wasps and pupae (*n* = 40 each) were placed in finger-shaped glass tubes, and the tube orifices were sealed with cotton wool. The tubes were kept under natural light. After pupae were naturally injected and paralysed by wasps, they were removed from the tubes and kept at 27 ± 1 °C and 85 % relative humidity (RH).

#### Venom injection

The prepared venom was diluted with PBS to a concentration of 0.3 venom reservoir equivalents [VRE, one VRE was defined as the supernatant from one torn venom reservoir in 1 µL PBS; pupae could be complete paralytic when injected with 0.3 VRE (Zhuo et al. 2013a)], and the diluent (1 μL) was injected into the abdomen of each pupa. Pupae were injected in groups of 40 individuals, and the same numbers of pupae were injected with PBS as a control. The injected pupae were kept at 27 ± 1 °C and 85 % RH.

### Methods

Pupae that had been injected with venom and PBS and kept for 1–6 and 10 days (some of the injected pupae would die after 10 days) were used as treatment groups in the subsequent experiments respectively. Pupae that had been parasitized (naturally injected) and non-parasitized and kept for 1–6 and 10 days were used as controls respectively. All pupae from 1 to 6 days were used for biochemical analyses and pupae at 10 days were used for observation of the fat body. All treatments were measured 3 times by using 40 pupae for each treatment and each nutrient, except analysis of fat-body protein and observation of fat body.

#### Sample pre-treatment

Sample pre-treatment followed methods described by Tan et al. (2003). We added 4 mL 0.15 mol L^−1^ perchloric acid to 1 g *T. molitor* pupae, and the pupae were ground to a homogenate. Then, 6 mL 0.15 mol L^−1^ perchloric acid was used to wash the mortar four times, and this solution was transferred into a centrifuge tube and centrifuged at 5000×*g* for 20 min. The supernatant was collected and filtered into a 25-mL volumetric flask. Distilled water was added to constant volume, and this mixture was used for sugar extraction.

#### Trehalose content

Trehalose content followed methods described by Tan et al. (2003). Sugar extract (1 mL) was added to 2 mL 0.075 mol L^−1^ sulfuric acid and the mixture was heated in a water bath for 10 min, 60 °C. While the mixture was cooling, 2 mL 30 % KOH was added, and the solution was boiled for 20 min to destroy reducing sugars. One milliliter of this solution was then added to 4 mL anthrone reagent (1.0 g anthrone dissolved in 1 L 95 % concentrated sulfuric acid) and mixed well. The mixed solution was boiled in a water bath for 10 min. While the solution cooled, the OD_620_ value was determined to calculate trehalose content.

A trehalose standard curve was prepared using standard (200 µg mL^−1^) glucose solution (0, 0.1, 0.2, 0.3, 0.4, 0.5, 0.6, and 0.7 mL). Water was added to each tube to bring the volume to 1 mL. Anthrone reagent (4 mL) was then added to each tube and mixed well, and the tubes were heated for 10 min. The OD_620_ value was measured while the solution was cooling. A standard curve was drawn with sugar content as the abscissa and OD_620_ value as the ordinate.

#### Glycogen content

Glycogen content was measured by adding 2 mL sugar extract, 6 mL 95 % ethanol, and 3 drops saturated Na_2_SO_4_ solution to a centrifuge tube. The solution was mixed well, left to stand for 3 days at 4 °C, and then centrifuged at 5000×*g* for 15 min. The supernatant was discarded and the residual liquid was briefly heated at 70 °C to remove ethanol. The precipitate was removed and dissolved in water; the dissolved solution was transferred into a 10-mL volumetric flask, and distilled water was added to constant volume and mixed well. One milliliter of this solution was added to 2 mL 0.075 mol L^−1^ sulfuric acid, and the mixture was heated in a water bath for 10 min. While the mixture cooled, 2 mL 30 % KOH was added, and this solution was boiled for 20 min to destroy reducing sugars. One milliliter of the boiled solution was added to 4 mL anthrone reagent and mixed well, and the mixed solution was boiled in a water bath for 10 min. The OD_620_ value was determined while the solution was cooling, for calculation of glycogen content.

A standard glycogen curve was prepared as described for trehalose in “Glycogen content” section.

#### Reducing sugar content

Reducing sugar content was determined by adding 1 mL sugar extract, 0.5 mL 3,5-dinitrosalicylic acid reagent, 6.3 g 3,5-dinitrosalicylic acid, and 262 mL 2 mol L^−1^ NaOH to a hot solution of 182 g potassium sodium tartrate dissolved in 500 mL water. Then, 5 g recrystallized phenol and 5 g Na_2_SO_3_ were dissolved in the solution and mixed well. As the solution was cooling, it was transferred to a 1000-mL volumetric flask, and distilled water was added to constant volume and mixed well. The yellow solution was stored in a brown bottle for 7 days and then heated in boiling water for 5 min. While it cooled, 4 mL double-distilled water (DDW) was added to the solution and mixed well, and the OD_540_ value was measured to determine the content of reducing sugar.

A standard was prepared using 0, 0.2, 0.3, 0.4, 0.5, and 0.6 mL standard glucose solution (200 µg mL^−1^). Water was added to each tube to bring the volume to 1 mL. Then, 0.5 mL 3,5-dinitrosalicylic acid reagent was added to each tube and mixed well, and the solution was heated for 5 min. While the solution cooled, 4 mL water was added to each tube and mixed well, and the OD_540_ value was measured. A standard curve was drawn with glucose content as the abscissa and OD_540_ value as the ordinate.

#### Analysis of hemolymph protein

Hemolymph collection (piercing method) followed methods described by Gäde et al. (2007). Hemolymph protein was analyzed by centrifuging 5 µL pupal hemolymph at 1000×*g* for 10 min to remove blood cells. The supernatant was collected to determine protein contents by electrophoresis according to Bradford (1976). The treatment was measured 3 times by using different pupae. We used sodium dodecyl sulfate polyacrylamide gel electrophoresis (SDS-PAGE). The concentrations of spacer and separating gels were 5 and 12 % respectively. Before appearance of the indicator in the separating gel, the electrical current was set at 5 mA; when the indicator appeared, the electrical current was set at 10 mA. Electrophoresis was terminated when the indicator was 1 cm from the bottom of the gel. The gel was then stained by Coomassie brilliant blue R250, scanned, and analyzed.

#### Analysis of fat-body protein

The pupal fat body was cleaned three times by PBS and dried using filter paper. The fat was weighed, ground, and centrifuged at 10,000×*g* for 10 min. The supernatant consisted of soluble extract of fat-body protein, which was determined as described for hemolymph protein (“Analysis of hemolymph protein” section).

#### Analysis of hemolymph lipid droplets

Hemolymph lipid droplets were analyzed according to Nakamatsu and Tanaka (2003). Pupal hemolymph (5 µL) was centrifuged at 1000×*g* for 10 min, and the supernatant was extracted by chloroform for 5 min. Then, 0.5 mL concentrated sulfuric acid was added and the solution was heated in boiling water for 10 min. After cooling to room temperature, 1 mL glutaraldehyde solution was added and the solution was stained for 30 min. Finally, the OD_547_ value was determined to calculate the content of hemolymph lipid droplets. A standard curve was prepared using cholesterol (2.5 mg mL^−1^ carbinol, concentration range 0–50 mg mL^−1^).

#### Analysis of fat-body lipid droplets

The pupal fat body was cleaned by PBS three times, dried using filter paper, freeze-dried, and weighed. The fat body was then homogenized by 0.5 mL chloroform and centrifuged at 1000×*g* for 10 min, and the supernatant was collected to determine the content of fat-body lipid droplets as described for hemolymph (“Analysis of hemolymph lipid droplets” section).

#### Observation of fat body

The complete or broken fat body was carefully removed under a dissecting microscope, stained by 0.1 % Sudan IV solution, and photographed with an camera system (Gel Doc XR, Bio-Rad, USA) for observation.

### Statistical analysis

All data were analyzed by one-way analysis of variance (ANOVA) and Duncan’s multiple-range tests, and all analyses were performed in SPSS 18.0 (SPSS Inc., Chicago, IL).

## Results

### Protein content

Each venom sac of *S. sichuanensis* Xiao contained approximately 3.078 ± 0.351 μg protein, and the protein concentration of 0.3 VRE was approximately 0.923 ± 0.105 μg μL^−1^.

### Changes in nutrients

#### Trehalose content

**Fig. 1:**
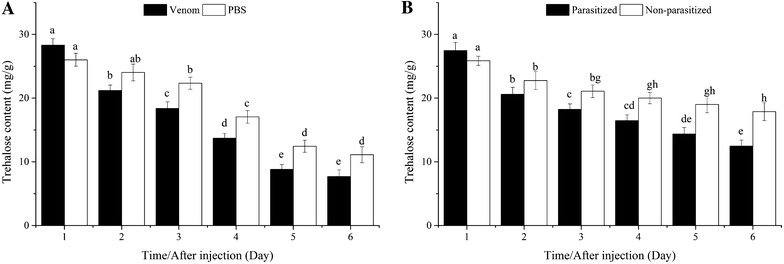
Comparison of trehalose content between venom-injected and phosphate-buffered saline (PBS)-injected pupae (**A**), parasitized and non-parasitized pupae (**B**). *Different lowercase* indicate significant differences (*P* < 0.05) among treatments, the same below

Trehalose content in hosts injected with venom or PBS decreased gradually and significantly from the first to the fifth day, after which no differences occurred. No significant differences were found between venom- and PBS-injected pupae (*P* > 0.05) treated on the first or second day. However, trehalose content was significantly lower in venom- than in PBS-injected pupae from the third to the sixth day (Fig. 1A).

Trehalose content in parasitized and non-parasitized hosts declined significantly from the first to the fifth day. After the third day, trehalose content was significantly lower in parasitized than in non-parasitized pupae (Fig. 1B).

#### Glycogen content

**Fig. 2:**
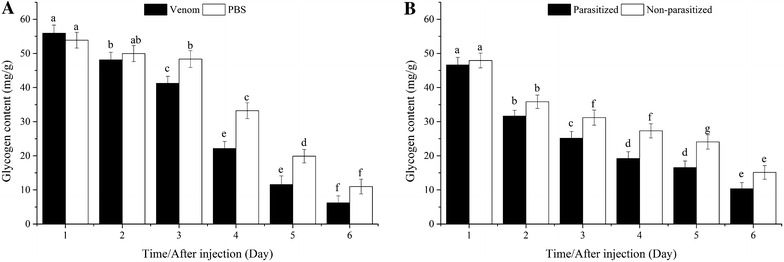
Comparison of glycogen content between venom-injected and phosphate-buffered saline (PBS)-injected pupae (**A**), parasitized and non-parasitized pupae (**B**)

In hosts injected with venom or PBS, glycogen content decreased significantly from the second to sixth day. From the third day, glycogen content was significantly lower in venom-injected than in PBS-injected pupae (Fig. 2A).

Parasitism (natural injection): When hosts were parasitized or non-parasitized, the Glycogen content decreased significantly from the first to the sixth day in both parasitized and non-parasitized hosts, and was significantly lower in parasitized pupae from the third day onward (Fig. 2B).

#### Reducing sugar content

**Fig. 3:**
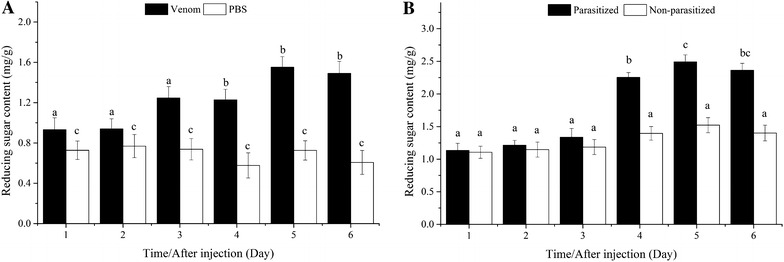
Comparison of reducing sugar content between venom-injected and phosphate-buffered saline (PBS)-injected pupae (**A**), parasitized and non-parasitized pupae (**B**)

Reducing sugar content increased significantly by the fourth day in venom-injected hosts, but did not change significantly in PBS-injected pupae (*P* > 0.05). Venom-injected pupae contained significantly higher quantities of reducing sugars than PBS-injected pupae from the first day onward (Fig. 3A).

Reducing sugar content increased significantly in parasitized hosts by the fourth day, but did not change significantly in non-parasitized pupae (*P* > 0.05). Parasitized pupae contained significantly higher quantities of reducing sugars than non-parasitized pupae from the fourth day onward (Fig. 3B).

#### Hemolymph protein

**Fig. 4:**
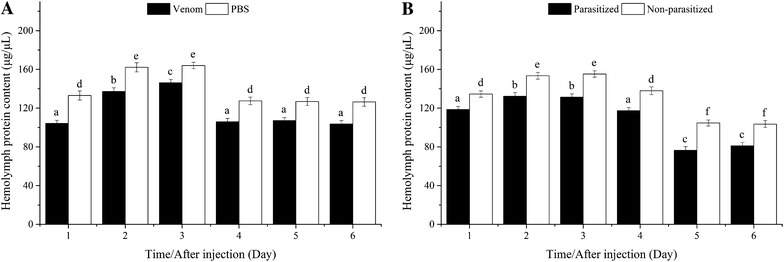
Comparison of hemolymph protein content between venom-injected and phosphate-buffered saline (PBS)-injected pupae (**A**), parasitized and non-parasitized pupae (**B**)

Hemolymph protein content increased and then declined significantly in hosts injected with venom or PBS. Venom-injected pupae contained significantly lower quantities of hemolymph protein than did PBS-injected pupae at all time points (Fig. 4A).

Hemolymph protein content increased significantly from the first to the third day, and then declined significantly, in both parasitized and non-parasitized hosts. Parasitized pupae contained significantly lower quantities of hemolymph protein than did non-parasitized pupae at all time points (Fig. 4B).

**Fig. 5:**
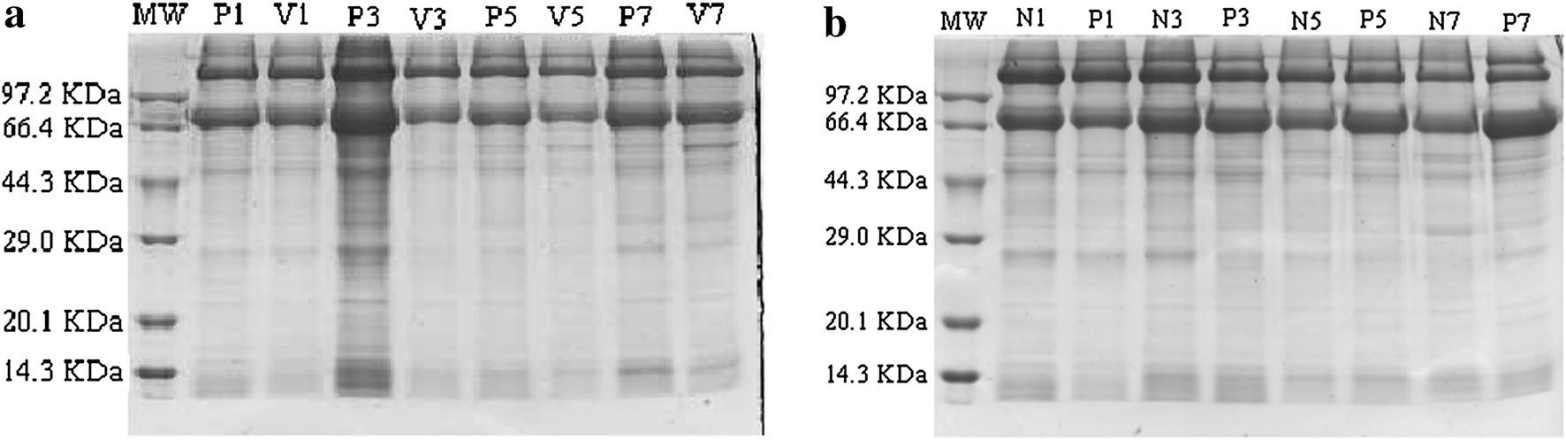
Sodium dodecyl sulfate–polyacrylamide gel electrophoresis (SDS-PAGE) electrophoretogram showing components of hemolymph protein for venom-injected and phosphate-buffered saline (PBS)-injected pupae (**a**), parasitized and non-parasitized pupae (**b**). *Note*: MW, protein molecular weight marker; *lanes P1*, *P3*, *P5*, and *P7* show protein extracted from PBS-injected pupae on days 1, 3, 5, and 7, respectively; *lanes N1*, *N3*, *N5*, and *N7* show protein extracted from non-parasitized pupae on days 1, 3, 5, and 7, respectively; *lanes V1*, *V3*, *V5*, and *V7* show protein extracted from venom-injected pupae on the same days; *lanes P1*, *P3*, *P5* and *P7* show protein extracted from parasitized pupae on the same days

The electrophoresis results showed that the constituents of hemolymph protein were similar between venom- and PBS-injected pupae (Fig. 5a) and between parasitized and non-parasitized pupae (Fig. 5b). This demonstrated that *S. sichuanensis* did not cause hydrolysis of hemolymph protein in parasitized pupae, and it confirmed the absence of narrow-spectrum parasitism peptides, which are secreted by certain idiobiont ectoparasitoid wasps.

#### Fat-body protein

**Fig. 6:**
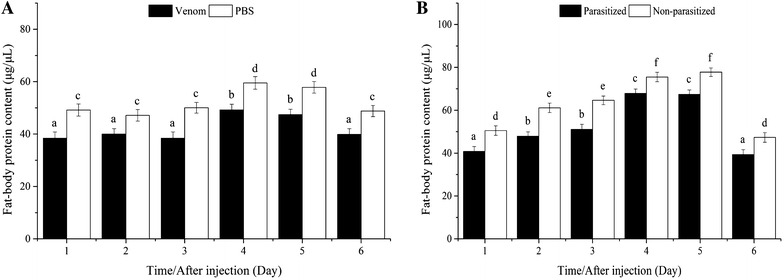
Comparison of fat-body protein content between venom-injected and phosphate-buffered saline (PBS)-injected pupae (**A**), parasitized and non-parasitized pupae (**B**)

Fat-body protein content increased significantly after the third day, and then declined after the fifth day, in venom- and PBS-injected pupae. Venom-injected pupae contained significantly less fat-body protein than did PBS-injected pupae at all time points (Fig. 6A).

Fat-body protein content increased significantly through the fifth day in parasitized and non-parasitized hosts, and was consistently lower in parasitized pupae (Fig. 6B).

#### Hemolymph lipid droplets

**Fig. 7:**
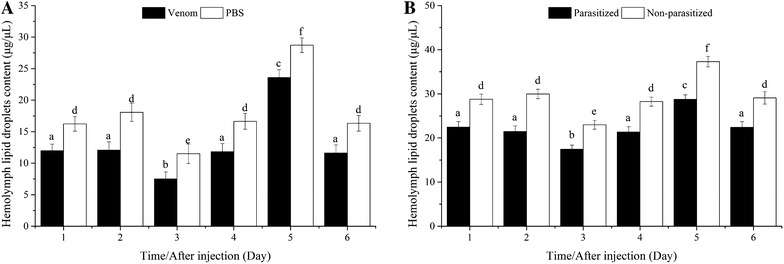
Comparison of hemolymph lipid droplet contents between venom-injected and phosphate-buffered saline (PBS)-injected pupae (**A**), parasitized and non-parasitized pupae (**B**)

The content of hemolymph lipid droplets decreased significantly after the second day in all hosts, and was significantly lower in hosts injected with venom (Fig. 7A) and in parasitized pupae (Fig. 7B) at all time points.

#### Fat-body lipid droplets

**Fig. 8:**
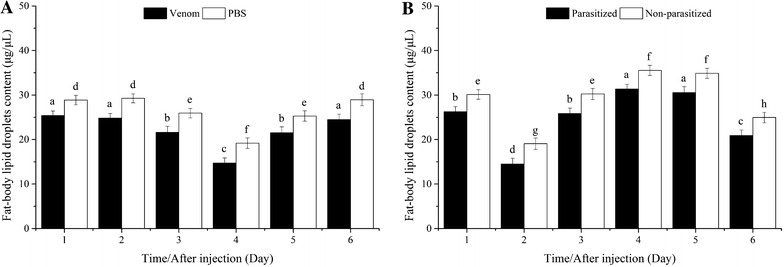
Comparison of fat-body lipid droplet content between venom-injected and phosphate-buffered saline (PBS)-injected pupae (**A**), parasitized and non-parasitized pupae (**B**)

Fat-body lipid droplets declined and then increased significantly from the fourth day, and were consistently and significantly lower in venom-injected pupae (Fig. 8A). The patterns of change in fat-body lipid droplets were similar in parasitized and non-parasitized pupae, with significantly lower contents in the parasitized hosts (Fig. 8B).

#### Fat-body observation

**Fig. 9:**
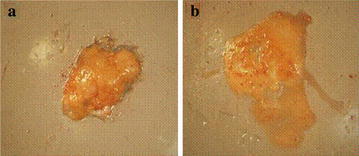
Fat body of venom-injected pupa (**a**) and parasitized pupa (**b**) on day 10

Pupal fat bodies were dyed orange with Sultan-IV solution. Fat bodies of venom-injected pupae were complete, semi-solid, and massive (Fig. 9a); those of parasitized pupae were incomplete, disintegrated into granules, and mixed with hemolymph (Fig. 9b).

## Discussion

To explore the regulatory mechanism of parasitic wasps on host physiology and biochemistry, we examined the effects of venom injection and parasitism by *S. sichuanensis* on *T. molitor* pupae. Our findings showed that this wasp could regulate host nutritional content, although venom and parasitism had significantly different effects on nutritional content regulation.

Host nutritional content was regulated by adult *S. sichuanensis* to create a suitable environment for growth and development of wasp offspring. Mechanisms for controlling host nutrient levels differ among wasp species, and among hosts for a given wasp species (Webb and Luckhart 1994). Parasitic systems are complex. For example, carbohydrate, protein, and lipid content can be consistently increased or decreased, or they can be up- and down-regulated over time (Cônsoli et al. 2005; Kaeslin et al. 2005; Nguyen et al. 2008).

Venom of *S. sichuanensis* can cause paralysis, bacteriostasis, inhibited exuviation, and delayed melanization in its hosts (Zhuo et al. 2013a, b). The wasp cannot feed on the host until host immune activity is weakened or lost. Parasitism and venom can suppress host immune activity while also regulating host nutritional content. In both parasitized and non-parasitized hosts, the levels of glycogen and trehalose (days 1–6), hemolymph protein (days 3–6), were decreased significantly; the levels of fat-body protein was increased (days 2–5) at beginning, but returned to the level shown on day 1; the levels of hemolymph lipid droplets was decreased (days 2–3) at beginning, but significant increased from days 3 to 5, and then returned to the level shown on day 1; the levels of fat-body lipid droplets was decreased (days 1–2) at beginning, but significant increased from days 2 to 5, and then decreased to the level between day 1 and day 3; the levels of reducing sugar content (days 3–6) was increased significantly. These findings confirmed that parasitism and venom caused storage sugars (glycogen and trehalose) to decompose into reducing sugars, and hemolymph protein to break down into free amino acids (Tan et al. 2003). This indicates that *S. sichuanensis* mainly feeds on reducing sugars and free amino acid, but host pupae have immunity, the nutritional level was wavy. In addition, changes in fat-body protein and lipids, as well as the hydrolysis of the fat body into granules, demonstrated that the wasp mainly fed on fat bodies from both the hemolymph and tissues of the host. Wasp-induced catabolism of macromolecules (sugars and proteins) could cause a significant decrease in carbohydrate (trehalose, glucose) contents. Parasitized hosts cannot maintain normal osmotic blood pressure, and these hosts die when their sugar contents are depleted. Idiobiont and koinobiont parasitoid wasps let hosts live and die respectively during the parasitic period; ectoparasitoid and endoparasitiod are wasps that parasitic on and in host respectively. Our findings confirmed that *S. sichuanensis* is a typical idiobiont ectoparasitoid wasp, and that host nutrient regulation by idiobiont and koinobiont wasps is similar.

There were some significant differences in host nutritional content between venom- and PBS-injected pupae. When hosts were injected with venom or PBS, the contents of lipid droplets (hemolymph and fat body) decreased at first but increased later and returned to initial levels. The contents of storage sugars (glycogen and trehalose) were significantly decreased, but reducing sugar contents increased significantly. Protein contents (hemolymph and fat body) increased at first, but then returned to initial levels. The effects of venom on glycogen, trehalose, reducing sugar, hemolymph protein, and hemolymph lipids were similar to the effects of parasitism. However, the fat bodies of pupae that were injected with venom could not be disintegrated into granules. It might confirm that factors other than venom alone regulate host nutritional content, such as digestive enzymes in wasp saliva. Furthermore, there were strong similarities between these two treatments: both parasitism and venom injection caused breakdown of storage sugars (glycogen and trehalose) into reducing sugars, and also reductions in protein and lipid content. The strong similarities between the two treatments suggest that venom injection is a major factor responsible for changes in host nutrient content.

Moreover, the effects of venom were faint and transitory for host sugar and protein contents, but were important for hemolymph lipid content. These findings suggested that when host pupae were parasitized or injected with venom, lipid catabolism might be accelerated. In addition, host pupae that were parasitized or injected with venom could not molt successfully as a result of changes in lipid content, because ecdysone insect prohormone ecdysone is composed of lipid (Askew and Shaw 1986). Those results (hemolymph lipid and fat-body lipid decreased) were similar to findings described by Rivers and Denlinger (1995), who showed that hemolymph lipid and fat-body lipid decreased significantly in *Sarcodexia sternodontus* injected with venom of *Nasonia vitripennis*. Other studies have confirmed that some effects of parasitism on hosts are transitory and can be sustained for only a few days. Nakamatsu et al. (2001) showed that trehalose content increased at days 7–10 in *Pseudaletia separate* injected with venom and calyx fluid of *Cotesia kariyai*, and that host physiological and biochemical changes were also caused by ovarian proteins, teratocytes, and parasitic wasp secretions. The physiological and biochemical changes observed in our parasitic system might be caused by some of these factors.

Narrow-spectrum peptides are secreted primarily by koinobiont parasites, which can compete with their hosts for nutrients. Narrow-spectrum peptides are secreted by teratocytes, PDV, and wasp offspring; they can regulate the host immune system, growth, development, and feeding behavior (Shelby and Webb 1997; Cônsoli et al. 2005; Kaeslin et al. 2005). Some scholars propose that endoparasitoid wasps evolved into ectoparasitoids, because some regulatory mechanisms (e.g., inhibition of host immunity and regulation of host behavior) were similar between these wasps at different evolutionary stages, and ecto- and endoparasitoid wasps have other similarities (Zhuo et al. 2013a). It declared that idiobiont and koinobiont wasps include both ecto- and endoparasitoids. Ren et al. (2004) showed that *Tetrastichus* sp. was an idiobiont endoparasitoid wasp, and that its parasitized host pupa *Ostrinia furnacalis* remained undecomposed for 20 days after death (Ren et al. 2004). Our electrophoretic results showed that there were no significant differences in the composition of hemolymph protein from parasitized pupae and pupae injected with venom. The lack of narrow-spectrum peptides in *S. sichuanensis* demonstrated that it is a typical idiobiont ectoparasitoid wasp too.

## Conclusions

This study indicates that *S. sichuanensis* is a typical idiobiont ectoparasitoid wasp, and might feeds mainly on reducing sugars and free amino acids, thus the wasp could obtain energy more easily and grow and breed more easily. Lipids consumed by *S. sichuanensis* might come from hemolymph and host tissue. And, parasitized and non-parasitized *T. molitor* pupae had significant nutrient content differences; Venom- and PBS-injected *T. molitor* pupae also had significant differences in nutrient content. All above, it indicates that venom is a major factor that regulates host nutritional content.
